# Supplementary material for: Health Promoting School Interventions in Latin America: A Systematic Review Protocol on the Dimensions of the RE-AIM Framework
Source: Int J Environ Res Public Health. 2020 Jul 31;17(15):5558. doi: 10.3390/ijerph17155558 (PMC7432100; doi:10.3390/ijerph17155558)
Supplement: Supplementary file 1 [file ijerph-17-05558-s001.pdf]

International Journal of
Environmental Research and Public Health

MDPI

Table S1. PRISMA-P (Preferred Reporting Items for Systematic review and Meta-Analysis Protocols) 2015 checklist: recommended items to address in a systematic review protocol *.

Section and Topic	Item No	Checklist Item	Page
		**ADMINISTRATIVE INFORMATION**	
Title:			
Identification	1a	Identify the report as a protocol of a systematic review	01
Update	1b	If the protocol is for an update of a previous systematic review, identify as such	Not apply
Registration	2	If registered, provide the name of the registry (such as PROSPERO) and registration number	02 e 03
Authors:			
Contact	3a	Provide name, institutional affiliation, e-mail address of all protocol authors; provide physical mailing address of corresponding author	01
Contributions	3b	Describe contributions of protocol authors and identify the guarantor of the review	07
Amendments	4	If the protocol represents an amendment of a previously completed or published protocol, identify as such and list changes; otherwise, state plan for documenting important protocol amendments	Not apply
Support:			
Sources	5a	Indicate sources of financial or other support for the review	07
Sponsor	5b	Provide name for the review funder and/or sponsor	07
Role of sponsor or funder	5c	Describe roles of funder(s), sponsor(s), and/or institution(s), if any, in developing the protocol	07
		**INTRODUCTION**	
Rationale	6	Describe the rationale for the review in the context of what is already known	02
Objectives	7	Provide an explicit statement of the question(s) the review will address with reference to participants, interventions, comparators, and outcomes (PICO)	03
		**METHODS**	
Eligibility criteria	8	Specify the study characteristics (such as PICO, study design, setting, time frame) and report characteristics (such as years considered, language, publication status) to be used as criteria for eligibility for the review	03 e 04
Information sources	9	Describe all intended information sources (such as electronic databases, contact with study authors, trial registers or other grey literature sources) with planned dates of coverage	04 e 05
Search strategy	10	Present draft of search strategy to be used for at least one electronic database, including planned limits, such that it could be repeated	Table S1

Study records:			
Data management	11a	Describe the mechanism(s) that will be used to manage records and data throughout the review	05
Selection process	11b	State the process that will be used for selecting studies (such as two independent reviewers) through each phase of the review (that is, screening, eligibility and inclusion in meta-analysis)	05
Data collection process	11c	Describe planned method of extracting data from reports (such as piloting forms, done independently, in duplicate), any processes for obtaining and confirming data from investigators	05
Data items	12	List and define all variables for which data will be sought (such as PICO items, funding sources), any pre-planned data assumptions and simplifications	05
Outcomes and prioritization	13	List and define all outcomes for which data will be sought, including prioritization of main and additional outcomes, with rationale	06
Risk of bias in individual studies	14	Describe anticipated methods for assessing risk of bias of individual studies, including whether this will be done at the outcome or study level, or both; state how this information will be used in data synthesis	05
Data synthesis	15a	Describe criteria under which study data will be quantitatively synthesised	06
	15b	If data are appropriate for quantitative synthesis, describe planned summary measures, methods of handling data and methods of combining data from studies, including any planned exploration of consistency (such as I^2, Kendall's τ)	06
	15c	Describe any proposed additional analyses (such as sensitivity or subgroup analyses, meta-regression)	06
	15d	If quantitative synthesis is not appropriate, describe the type of summary planned	06
Meta-bias(es)	16	Specify any planned assessment of meta-bias(es) (such as publication bias across studies, selective reporting within studies)	Not apply
Confidence in cumulative evidence	17	Describe how the strength of the body of evidence will be assessed (such as GRADE)	06

* It is strongly recommended that this checklist be read in conjunction with the PRISMA-P Explanation and Elaboration (cite when available) for important clarification on the items. Amendments to a review protocol should be tracked and dated. The copyright for PRISMA-P (including checklist) is held by the PRISMA-P Group and is distributed under a Creative Commons Attribution Licence 4.0.

From: Shamseer L, Moher D, Clarke M, Ghersi D, Liberati A, Petticrew M, Shekelle P, Stewart L, PRISMA-P Group. Preferred reporting items for systematic review and meta-analysis protocols (PRISMA-P) 2015: elaboration and explanation. BMJ. 2015 Jan 2;349(jan02 1):g7647.

Table S2. Example of search terms, which will be used at Medline/Pubmed.

Groups	Search Terms
Population	child[MeSH Terms] OR adolescent[MeSH Terms] OR students[MeSH Terms] OR student[Text Word] OR students[Text Word] OR "young children"[Text Word] OR "young people"[Text Word] OR children[Text Word] OR adolescent[Text Word] OR adolescents[Text Word] OR young[Text Word] OR pupils[Text Word] OR Schoolage*[Text Word] OR schoolchild*[Text Word] OR schooler*[Text Word]
Context (Countries)	("Central America"[MeSH Terms] OR "South America"[MeSH Terms] OR Caribbean[MeSH Terms] OR "hispanic americans"[MeSH Terms] OR "latin america"[MeSH Terms] OR Aruba[MeSH Terms] OR "Caribbean Netherlands"[MeSH Terms] OR Curacao[MeSH Terms] OR "Sint Maarten"[MeSH Terms] OR "West Indies″[MeSH Terms] OR "Antigua and Barbuda"[MeSH Terms] OR Bahamas[MeSH Terms] OR Barbados[MeSH Terms] OR "British Virgin Islands"[MeSH Terms] OR Cuba[MeSH Terms] OR Dominica[MeSH Terms] OR "Dominican Republic"[MeSH Terms] OR Grenada[MeSH Terms] OR Guadeloupe[MeSH Terms] OR Haiti[MeSH Terms] OR Jamaica[MeSH Terms] OR Martinique[MeSH Terms] OR "Puerto Rico"[MeSH Terms] OR "Saint Kitts and Nevis"[MeSH Terms] OR "Saint Lucia"[MeSH Terms] OR "Saint Vincent and the Grenadines"[MeSH Terms] OR "Trinidad and Tobago"[MeSH Terms] OR "United States Virgin Islands"[MeSH Terms] OR Belize[MeSH Terms] OR Costa Rica[MeSH Terms] OR "El Salvador"[MeSH Terms] OR Guatemala[MeSH Terms] OR Honduras[MeSH Terms] OR Nicaragua[MeSH Terms] OR Panama[MeSH Terms] OR Argentina[MeSH Terms] OR Bolivia[MeSH Terms] OR Brazil[MeSH Terms] OR Chile[MeSH Terms] OR Colombia[MeSH Terms] OR Ecuador[MeSH Terms] OR "French Guiana"[MeSH Terms] OR Guyana[MeSH Terms] OR Paraguay[MeSH Terms] OR Peru[MeSH Terms] OR Suriname[MeSH Terms] OR Uruguay[MeSH Terms] OR Venezuela[MeSH Terms] OR Mexico[MeSH Terms]) OR ("Central America"[Text Word] OR "South America"[Text Word] OR Caribbean[Text Word] OR "hispanic americans"[Text Word] OR "latin america"[Text Word] OR Aruba[Text Word] OR "Caribbean Netherlands"[Text Word] OR Curacao[Text Word] OR "Sint Maarten"[Text Word] OR "West Indies″[Text Word] OR "Antigua and Barbuda"[Text Word] OR Bahamas[Text Word] OR Barbados[Text Word] OR "British Virgin Islands"[Text Word] OR Cuba[Text Word] OR Dominica[Text Word] OR "Dominican Republic"[Text Word] OR Grenada[Text Word] OR Guadeloupe[Text Word] OR Haiti[Text Word] OR Jamaica[Text Word] OR Martinique[Text Word] OR "Puerto Rico"[Text Word] OR "Saint Kitts and Nevis"[Text Word] OR "Saint Lucia"[Text Word] OR "Saint Vincent and the Grenadines"[Text Word] OR "Trinidad and Tobago"[Text Word] OR "United States Virgin Islands"[Text Word] OR Belize[Text Word] OR Costa Rica[Text Word] OR "El Salvador"[Text Word] OR Guatemala[Text Word] OR Honduras[Text Word] OR Nicaragua[Text Word] OR Panama[Text Word] OR Argentina[Text Word] OR Bolivia[Text Word] OR Brazil[Text Word] OR Chile[Text Word] OR Colombia[Text Word] OR Ecuador[Text Word] OR "French Guiana"[Text Word] OR Guyana[Text Word] OR Paraguay[Text Word] OR Peru[Text Word] OR Suriname[Text Word] OR Uruguay[Text Word] OR Venezuela[Text Word] OR Mexico[Text Word])

Context (school)	"Schools"[Mesh] OR School*[Text Word]
Health Promotion	"school health services"[MeSH Terms] OR “health education”[MeSH Terms] OR “health education”[Text Word] OR “Health Promotion”[MeSH Terms] OR “Health Promotion"[Text Word] OR “Preventive Health Services”[MeSH Terms] OR “school program”[Text Word] OR “health program”[Text Word] OR “primary prevention”[Text Word] OR “secondary prevention”[Text Word] OR preventive measure* OR preventative measure* OR “preventive care”[Text Word] OR “preventative care”[Text Word] OR "health policy"[Text Word] OR "health policy"[MeSH Terms] OR "Attitude to Health"[MeSH Terms] OR "health status"[MeSH Terms] OR "Health Behavior"[MeSH Terms] OR Life Style[MeSH Terms]
HPS Framework	((built[Text Word] OR social[Text Word] OR physical[Text Word]) AND (environment[Text Word] OR environmental[Text Word]) AND (intervention[Text Word] OR component[Text Word] OR strategy[Text Word] OR education[Text Word] OR change)) OR (("schools"[MeSH Terms] OR "schools"[Text Word] OR "school"[Text Word]) AND ("climate"[MeSH Terms] OR "climate"[Text Word] OR "ethos"[Text Word] OR "culture"[Text Word] OR "culture"[MeSH Terms])) OR ((family[Text Word] OR parents[Text Word] OR community[Text Word] OR neighborhood[Text Word]) AND (intervention[Text Word] OR component[Text Word] OR strategy[Text Word] OR education[Text Word] OR change [Text Word])) OR (("health"[MeSH Terms] OR "health"[Text Word]) AND ("curriculum"[MeSH Terms] OR "curriculum"[Text Word] OR "teaching"[MeSH Terms] OR "teaching"[Text Word] OR "education"[Text Word] OR "education"[MeSH Terms] OR “School Teachers"[MeSH Terms])) OR ("Health Promoting School"[Text Word] OR "School Health Promotion"[Text Word] OR "Health Promotion in School"[Text Word] OR "school-wide"[Text Word] OR schoolwide[Text Word] OR "whole school"[Text Word] OR “multi-component”[Text Word] OR “multicomponent”[Text Word] OR multi-strategy[Text Word] OR multistrategy[Text Word] OR multifacet*[Text Word] OR multi-facet*[Text Word] OR multi-level[Text Word] OR multilevel[Text Word])
Study Design (Intervention studies)	“controlled clinical trial"[Publication Type] OR "controlled clinical trials as topic"[MeSH Terms] OR "controlled clinical trial"[All Fields] OR “randomized controlled trial”[Publication Type] OR "randomized controlled trials as topic"[MeSH Terms] OR “random allocation”[MeSH Terms] OR “double-blind method”[MeSH Terms] OR “single-blind method”[MeSH Terms] OR “double blind study”[text word] OR “single blind study”[text word] OR “triple blind study”[text word] OR (clinical* [Text Word] AND trial* [Text Word] OR ((randomised[Text Word] OR randomized[Text Word] OR randomly[Text Word]) AND (trial*[Text Word] OR group*[Text Word])) OR trial*[Title] OR “non-randomized controlled trial”[text word] OR "non-randomized controlled trials as topic"[MeSH Terms] OR “nonrandomized controlled trial”[text word] OR “non-randomized trial”[text word] OR “intervention study”[text word] OR “intervention studies”[text word] OR “intervention program”[text word] OR "intervention trial"[text word] OR "Comparative Study"[Publication Type] OR "Evaluation Study"[Publication Type] OR “follow-up studies”[MeSH Terms] OR “prospective studies”[MeSH Terms] OR "longitudinal studies"[MeSH Terms] OR “cross-over studies”[MeSH Terms] OR “interrupted time series

	analysis”[MeSH Terms] OR “quasiexperimental”[Text Word]) OR “quasi experimental”[Text Word] OR “pseudo experimental”[Text Word] OR "Before-After Studies"[text word] OR "Before-After Study"[text word] OR “Controlled Before-After Studies”[MeSH Terms] OR “Outcome Assessment, Health Care"[MeSH Terms]

Table S3. Example of search terms, which will be used at Lilacs, Scielo, BTDT and ReBEC.

**Grupos**	**Termos de busca**
Population	(tw:(adolescent*)) OR (tw:(adolescência)) OR (tw:(estudant*)) OR (tw:(crianc*)) OR (tw:(jovens)) OR (tw:(jovem)) OR (tw:(alun*)) OR (tw:(escolar*))
Context (Countries)	(tw:("América Central")) OR (tw:("América do Sul")) OR (tw:(" Caribe")) OR (tw:("Hispano-Americanos")) OR (tw:("America latina")) OR (tw:(Aruba)) OR (tw:("Países Baixos Caribenhos")) OR (tw:(Curaçao)) OR (tw:("São Martinho")) OR (tw:("Países Baixos")) OR (tw:("Índias Ocidentais")) OR (tw:("Antígua e Barbuda")) OR (tw:(Bahamas)) OR (tw:(Barbados)) OR (tw:("Ilhas Virgens Britânicas")) OR (tw:(Cuba)) OR (tw:(Dominica)) OR (tw:("República Dominicana")) OR (tw:(Granada)) OR (tw:(Guadalupe)) OR (tw:(Haiti)) OR (tw:(Jamaica)) OR (tw:(Martinica)) OR (tw:("Porto Rico")) OR (tw:("São Cristóvão e Névis")) OR (tw:("Santa Lúcia")) OR (tw:("São Vicente e Granadinas")) OR (tw:("Trinidad e Tobago")) OR (tw:("Ilhas Virgens Americanas")) OR (tw:(Belize)) OR (tw:("Costa Rica")) OR (tw:("El Salvador")) OR (tw:(Guatemala)) OR (tw:(Honduras)) OR (tw:(Nicarágua)) OR (tw:(Panamá)) OR (tw:(Argentina)) OR (tw:(Bolívia)) OR (tw:(Brasil)) OR (tw:(Chile)) OR (tw:(Colômbia)) OR (tw:(Equador)) OR (tw:("Guiana Francesa")) OR (tw:(Guiana)) OR (tw:(Paraguai)) OR (tw:(Peru)) OR (tw:(Suriname)) OR (tw:(Uruguai)) OR (tw:(Venezuela)) OR (tw:(México))
Context (school)	(tw:(escol*))
Health Promotion	(tw:("Serviços de Saúde Escolar")) OR (tw:("Educação em Saúde")) OR (tw:("Promoção da Saúde")) OR (tw:("Serviços Preventivos de Saúde")) OR (tw:("programa escolar")) OR (tw:("programa de saúde")) OR (tw:("Prevenção Primária")) OR (tw:("Prevenção Secundária")) OR (tw:(medida preventiva*)) OR (tw:("Medicina Preventiva")) OR (tw:("Política de Saúde")) OR (tw:("Atitude Frente a Saúde")) OR (tw:("Nível de Saúde")) OR (tw:("Comportamentos Relacionados com a Saúde")) OR (tw:("Estilo de Vida")) OR (tw:("saúde na escola"))
HPS Framework	(tw:((construído OR social OR físic*) AND (Ambiente) AND (intervenção OR componente OR estratégia OR educação OR mudança))) OR (tw:((escola*) AND (clima OR ética OR cultura OR família OR pais OR comunidade OR residência OR casa) AND (intervenção OR componente OR estratégia OR educação OR mudança))) OR (tw:(("Educação em saúde" OR "Ensino em saúde" OR "Currículo em Saúde" OR "Currículo de saúde"))) OR (tw:(("Escola Promotora de Saúde" OR "Promoção da Saúde na Escola" OR "multicomponente" OR "multi componente" OR "multi-estratégia" OR multifacetad* OR multi-nível)))

Study Design (Intervention studies)	(tw:("Ensaio Clínico Controlado")) OR (tw:("Ensaio Clínico Controlado Aleatório")) OR (tw:("Distribuição Aleatória")) OR (tw:("Método Duplo-Cego")) OR (tw:("Método Simples-Cego")) OR (tw:("Método Triplo-Cego")) OR (tw:("Ensaio Clínico")) OR (tw:("teste aleatório")) OR (tw:("grupo aleatório")) OR (tw:("grupo randomizado")) OR (tw:("Ensaios Clínicos Controlados não Aleatórios como Assunto")) OR (tw:("Ensaios controlados não randomizados")) OR (tw:("ensaio não randomizado")) OR (tw:("ensaio clínico")) OR (tw:("estudo de intervenção")) OR (tw:("programa de intervenção")) OR (tw:("julgamento da intervenção")) OR (tw:("Estudo comparativo")) OR (tw:("Estudo de avaliação")) OR (tw:(seguimentos)) OR (tw:("estudos prospectivos")) OR (tw:("estudos longitudinais")) OR (tw:("Estudos Cross-Over")) OR (tw:("Análise de Séries Temporais Interrompida")) OR (tw:(quasiexperimental)) OR (tw:(intervenção*)) OR (tw:(programa*)) OR (tw:(ensaio*)) OR (tw:(experiment*)) OR (tw:("estudo clínico"))
(Intervention studies)	tw:((tw:((tw:("Ensaio Clínico Controlado")) OR (tw:("Ensaio Clínico Controlado Aleatório")) OR (tw:("Distribuição Aleatória")) OR (tw:("Método Duplo-Cego")) OR (tw:("Método Simples-Cego")) OR (tw:("Método Triplo-Cego")) OR (tw:("Ensaio Clínico")) OR (tw:("teste aleatório")) OR (tw:("grupo aleatório")) OR (tw:("grupo randomizado")) OR (tw:("Ensaios Clínicos Controlados não Aleatórios como Assunto")) OR (tw:("Ensaios controlados não randomizados")) OR (tw:("ensaio não randomizado")) OR (tw:("ensaio clínico")) OR (tw:("estudo de intervenção")) OR (tw:("programa de intervenção")) OR (tw:("julgamento da intervenção")) OR (tw:("Estudo comparativo")) OR (tw:("Estudo de avaliação")) OR (tw:(seguimentos)) OR (tw:("estudos prospectivos")) OR (tw:("estudos longitudinais")) OR (tw:("Estudos Cross-Over")) OR (tw:("Análise de Séries Temporais Interrompida")) OR (tw:(quasi experimental)) OR (tw:(pseudo experimental)) OR (tw:("Estudos Antes-Depois")) OR (tw:("Estudos Controlados Antes e Depois")) OR (tw:("Avaliação de Resultados em Cuidados de Saúde")) OR (tw:(intervenção*)) OR (tw:(programa*)) OR (tw:(ensaio*)) OR (tw:(experiment*)) OR (tw:("estudo clínico")))) AND (tw:((tw:((construído OR social OR físic*) AND (ambiente) AND (intervenção OR componente OR estratégia OR educação OR mudança))) OR (tw:((escola*) AND (clima OR ética OR cultura OR família OR pais OR comunidade OR residência OR casa) AND (intervenção OR componente OR estratégia OR educação OR mudança))) OR (tw:(("Educação em saúde" OR "Ensino em saúde" OR "Currículo em Saúde" OR "Currículo de saúde"))) OR (tw:(("Escola Promotora de Saúde" OR "Promoção da Saúde na Escola" OR "multicomponente" OR "multi componente" OR "multi-estratégia" OR multifacetad* OR multi-nível))))) AND (tw:((tw:("Serviços de Saúde Escolar")) OR (tw:("Educação em Saúde")) OR (tw:("Promoção da Saúde")) OR (tw:("Serviços Preventivos de Saúde")) OR (tw:("programa escolar")) OR (tw:("programa de saúde")) OR (tw:("Prevenção Primária")) OR (tw:("Prevenção Secundária")) OR (tw:(medida preventiva*)) OR (tw:("Medicina Preventiva")) OR (tw:("Política de Saúde")) OR (tw:("Atitude Frente a Saúde")) OR (tw:("Nível de Saúde")) OR (tw:("Comportamentos Relacionados com a Saúde")) OR (tw:("Estilo de Vida")) OR (tw:("saúde na escolar")))) AND (tw:((tw:(escol*)))) AND (tw:((tw:("América Central")) OR (tw:("América do Sul")) OR (tw:("Região do Caribe")) OR (tw:("Hispano-Americanos")) OR (tw:("America latina")) OR (tw:(aruba)) OR (tw:("Países Baixos Caribenhos")) OR (tw:(curaçao)) OR (tw:("São Martinho")) OR (tw:("Países Baixos")) OR (tw:("Índias Ocidentais")) OR (tw:("Antígua e Barbuda")) OR (tw:(bahamas)) OR (tw:(barbados)) OR (tw:("Ilhas Virgens Britânicas")) OR (tw:(cuba)) OR (tw:(dominica)) OR (tw:("República Dominicana")) OR (tw:(granada)) OR (tw:(guadalupe)) OR (tw:(haiti)) OR (tw:(jamaica)) OR (tw:(martinica)) OR (tw:("Porto Rico")) OR (tw:("São Cristóvão

	e Névis")) OR (tw:("Santa Lúcia")) OR (tw:("São Vicente e Granadinas")) OR (tw:("Trinidad e Tobago")) OR (tw:("Ilhas Virgens Americanas")) OR (tw:(belize)) OR (tw:("Costa Rica")) OR (tw:("El Salvador")) OR (tw:(guatemala)) OR (tw:(honduras)) OR (tw:(nicarágua)) OR (tw:(panamá)) OR (tw:(argentina)) OR (tw:(bolívia)) OR (tw:(brasil)) OR (tw:(chile)) OR (tw:(colômbia)) OR (tw:(equador)) OR (tw:("Guiana Francesa")) OR (tw:(guiana)) OR (tw:(paraguai)) OR (tw:(peru)) OR (tw:(suriname)) OR (tw:(uruguai)) OR (tw:(venezuela)) OR (tw:(méxico)))) AND (tw:((tw:(adolescent*)) OR (tw:(adolescência)) OR (tw:(estudant*)) OR (tw:(crianc*)) OR (tw:("crianças pequenas")) OR (tw:(jovens)) OR (tw:(jovem)) OR (tw:(alun*)) OR (tw:(escolar*))))) AND (db:("LILACS") AND type_of_study:("clinical_trials" OR "cohort" OR "case_control"))

Table S4. Characteristics to be extracted related to implementation.

Category	Variable
Characteristics of the studies	Reference (Author and Year)
	Type of Study
	Country of the Study
	Year of the Intervention
	Duration of the Intervention (in months)
	Place of Publication
Description of the Population	Sample Selection Method
	Inclusion Criteria
	Exclusion Criteria
	N of subjects in each group (% by sex)
	Age group or mean age (standard deviation)
	N of schools in each group
Description of the Intervention	Was the HPS-WHO theory reported as a theoretical basis for the intervention?
	Description of changes or adaptations in the curriculum (What? How? Where? How much? Who performed it?)
	Description of changes or adaptations in the school environment (What? How? Where? How much? Who performed it?)
	Description of changes or adaptations in the relationship with family/community (What? How? Where? How much? Who performed it?)
	Was there any way of evaluating the implementation of the intervention (yes/no)

Description of implementation evaluation procedures	Which approach (qualitative, quantitative, mixed methods) was used to evaluate the implementation?
	What tools were used to evaluate the implementation?
	Who conducted the evaluation of the implementation?
	Which dimensions of the implementation were evaluated?
	How often and at what times was the evaluation of the implementation conducted?
	What criteria or tools were used to evaluate the implementation?
1st HPS Dimension - Health Education and Curriculum	Reports on Acceptability
	Reports on Appropriateness
	Reports on Feasibility
	Reports on Adoption
	Reports on Fidelity
	Reports on Penetration
	Reports on Sustainability
	Reports on Costs
2nd HPS Dimension - Social and/or Physical Environment of Schools to Health Promotion and Well-Being	Reports on Acceptability
	Reports on Appropriateness
	Reports on Feasibility
	Reports on Adoption
	Reports on Fidelity
	Reports on Penetration
	Reports on Sustainability
	Reports on Costs
3rd HPS Dimension - Engagement with Communities and Families	Reports on Acceptability
	Reports on Appropriateness
	Reports on Feasibility
	Reports on Adoption
	Reports on Fidelity
	Reports on Penetration
	Reports on Sustainability
	Reports on Costs

Source: Adapted from Moore et al. (2015) and Proctor et al. (2011).

Table S5. Characteristics to be extracted related to RE-AIM.

RE-AIM Category	Variable
Characteristics of the studies	Reference (Author and Year)
	Type of Study
	Country of the Study
	Year of the Intervention
	Duration of the Intervention
	Place of Publication or Indexation
Reach	1.1 Description of the target population
	1.2 Method to identify target population
	1.3 Inclusion criteria
	1.4 Exclusion criteria
	1.5 Participation rate
Effectiveness/Efficacy	2.1 Results of the primary outcomes
	2.2 Intent to treat or present in the follow-up
	2.3 Measures of quality of life
	2.4 Dropout rate (at program termination)
Adoption	3.1 Local participation rate
	3.2 Description of the place where the program was developed
	3.3 Inclusion/exclusion criteria of the team members
	3.4 Participation rate of the team members
	3.5 Method of team members identification
	3.6 Level of knowledge from team members
Implementation	4.1 Number/frequency/duration of contacts
	4.2 Measure to which the protocol was executed as expected
	4.3 Cost measures
Maintenance	5.1 Program continuity
	5.2 Evaluation of results after program termination (duration of follow-up)
	5.3 Individual dropout rate (during follow-up)

Source: Adapted from Brito et al. (2018).
